# Three-dimensional visualization of an unusual pulmonary lymphoproliferation after COVID-19

**DOI:** 10.1177/2050313X251395978

**Published:** 2025-11-20

**Authors:** Maximilian Ackermann, Jakob Reichmann, Jakob Frost, Tobias Welte, Stijn E. Verleden, Tim Salditt, Danny D. Jonigk, Reinhard Pabst

**Affiliations:** 1 Institute of Pathology, University Clinics Aachen, RWTH University of Aachen, Germany; 2 Institute of Pathology and Molecular Pathology, Helios University Clinic Wuppertal, University of Witten/Herdecke, Germany; 3 Institute of Anatomy, University Medical Center of the Johannes Gutenberg-University Mainz, Germany; 4 Institute for X-Ray Physics, University of Göttingen, Germany; 5 Department of Respiratory Medicine, Hannover Medical School, Germany; 6 German Center for Lung Research (DZL), Biomedical Research in Endstage and Obstructive Lung Disease Hannover (BREATH), Germany; 7 University of Antwerp, Antwerpen, Belgium; 8 Institute of Immunomorphology, Centre of Anatomy, Medical School Hannover, Germany

**Keywords:** bronchus-associated lymphoid tissue, BALToma, SARS-CoV2, multiscale X-ray phase-contrast computed tomography

## Abstract

The following case report details the case of a 40-year-old Caucasian patient who presented with dyspnea following a serologically confirmed mild-to-severe pulmonary infection with SARS-CoV-2. Chest computer tomography revealed a solitary ground-glass pulmonary nodule in the lower right lobe, measuring 2.1 cm in diameter. Video-assisted thoracoscopic surgery wedge resection revealed well-circumscribed lymphoid aggregates adjacent to the round, smaller airways, bronchioles, and blood vessels. IgKappa B exhibited a monoclonal polyclonal pattern, in contrast to the behavior exhibited by IgKappa A and IgLambda. In the following discussion, the lymphoid lesion was considered in the context of lymphoid hyperplasia, accompanied by an early infiltration of low-grade extranodal B cell lymphoma of the bronchus-associated lymphoid tissue (BALToma).

## Case presentation

**Figure 1.**
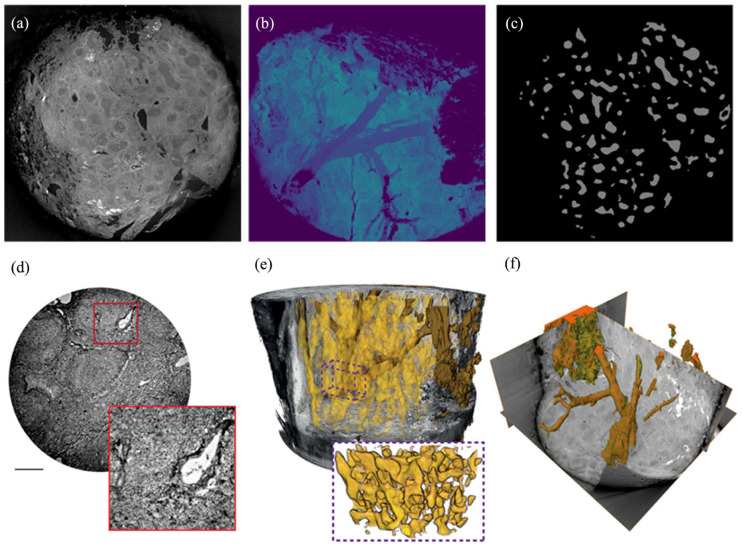
Paraffin-embedded lung tissue was analyzed by multiscale XPCT. (a–c) XPCT-scan could reveal the peribronchial, nodular infiltration of a lymphoid aggregates with GCs (rendered GCs in c) in the BALT. (d) XPCT scan demonstrates the perivascular spreading of lymphoid cells within and surrounding BALT. (e) Segmentation of vascularity is characterized by altered severely abnormal-appearing blood vessels (yellow) which are directly linked to the upstream larger blood vessels (f). XPCT: X-ray phase-contrast computed tomography; GCs: germinal centers; BALT: bronchus-associated lymphoid tissue.

**Figure 2.**
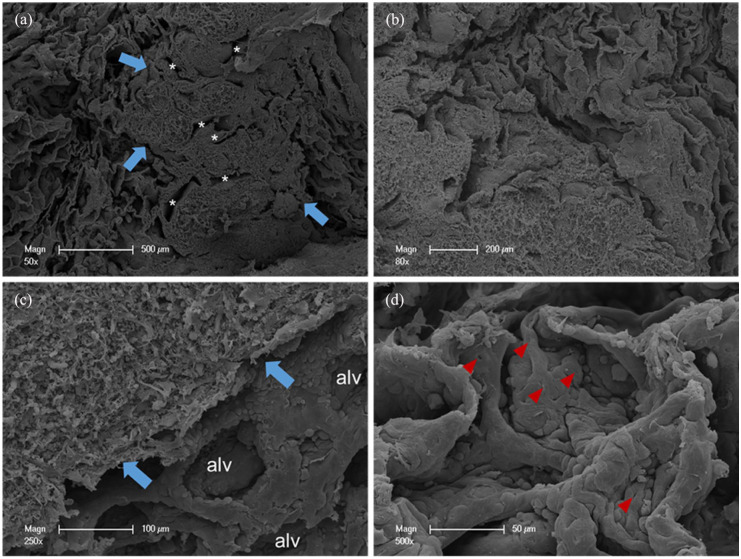
(a–c) Scanning electron micrographs of resected lung tissue illustrated the substantial infiltration of a peribronchiolar and perivascular (white asterisk) lymphoid aggregates (blue arrows) and prominent vascular proliferations with intussusceptive pillars (d, arrowheads). alv: alveolus.

**Figure 3.**
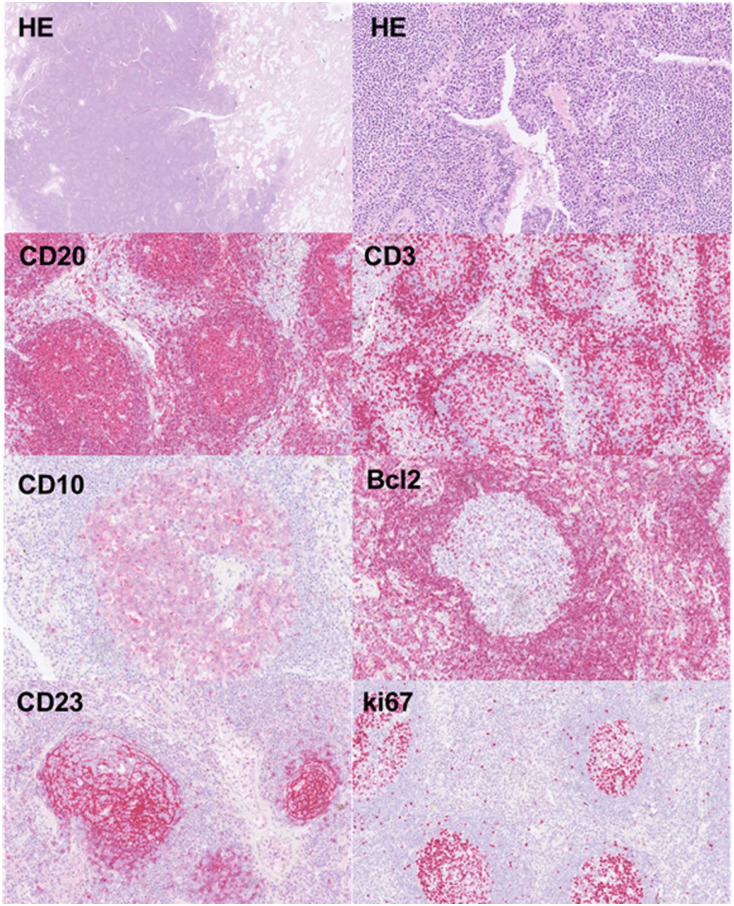
Immunohistochemical assessment of the peribronchial and perivascular lymphoid infiltrate revealed strong CD20- and Bcl2-positivity of germinal centers (CD20, CD23) and the paracortical lymphoid neoplasia with scattered T cells (CD3). Proliferation was slightly elevated in the paracortical zone.

A 40-year-old Caucasian woman with no smoking history was admitted with dyspnea and complaints of fatigue. After she had been afflicted by a serologically confirmed mild-severe pulmonary SARS-CoV2-infection 4 weeks ago. It was found that no other active or past infectious diseases, such as tuberculosis or influenza, could be clinically detected. A review of the patient’s medical history revealed no previous illnesses or autoimmune diseases. Furthermore, typical serum markers for lymphoma were not elevated. Physical examination, complete blood count, and liver and renal function tests were totally within physiological limits. Chest computer tomography revealed a subpleural, single ground-glass pulmonary nodule in the right lower lobe with the size of 2.1 × 2 × 1.6 cm. She underwent a video-assisted thoracoscopic surgery wedge resection of this suspicious pulmonary nodule. We analyzed the resected lung tissue by multiscale X-ray phase-contrast computed tomography (Figure 1) and scanning electron microscopy (Figure 2). Histopathological examination revealed well-circumscribed lymphoid aggregates adjacent to the round smaller airways, bronchioles, and blood vessels. Lung parenchyma with tumor-like interspersing of lymphoid tissue aggregates was seen, with germinal centers with mantle zones of variable width (ki67, CD10, CD20, Bcl2). Large networks of follicular dendritic reticulum cells (CD23-positive) were repeatedly seen (Figure 3). In the interfollicular regions and high-endothelial venules (HEV), there were aggregates of small lymphocytes, often primarily B cells (CD20) mixed with abundant intermixed T cells (CD3 and CD5 positive) and some plasma cells and histiocytes. Cell proliferation index of the lymphoid aggregate was less than 5% (ki67). LEF1 was partially positive, while cyclin D1 was negative. The morphological and immunohistochemical findings were interpreted as lymphoid hyperplasia of bronchus-associated lymphoid tissue (BALT), subject to confirmation by molecular analysis. Heteroduplex analysis was used to examine the B lymphocytes for the presence of polyclonal and clonal populations. For this purpose, three multiplex PCRs for heavy chains (IgH) and three multiplex PCRs for the light chains (Ig–κ/λ) and then analyzed by capillary gel electrophoresis. Immunoglobulin heavy chain (IgH) gene rearrangement study revealed polyclonality of the lymphoid lesion, whereas the analysis of IgKappa B was monoclonal, polyclonal pattern in IgKappa A und IgLambda.

Despite the unusual nature of this B cell lymphoproliferation with repeated monoclonal protein bands in IgK, the differential diagnostic classification was challenging due to the inconclusive immune profile. The differential diagnosis of nodular lymphofollicular hyperplasia and partial infiltration by a marginal cell lymphoma was particularly relevant in this case. Other B cell lymphomas, such as SLL or mantle cell lymphoma, could be excluded with confidence. In addition, an association of a previous inflammation with COVID-19 disease seems conceivable. Taken together, the lymphoid lesion was interpreted as a lymphoid hyperplasia with an early infiltration of by a low-grade extranodal B cell lymphoma of the bronchus-associated lymphoid tissue (BALToma). The patient underwent extensive clinical and radiological staging procedures. Subsequent follow-ups will be conducted at regular intervals to monitor the patient’s condition.

## Discussion

Primary lymphomas of the lung are rare, whereas BALT lymphomas are the most common entity, accounting for approximately 75%–90% of all cases. The median age of presentation is 62 years. Females are more commonly affected, making up 55% of patients. Approximately 10% of patients suffer from autoimmune disease, 45% are smokers, while 19% have a known preexisting lung disease.^1^ The overall prognosis for patients with mucosa-associated lymphoid tissue (MALT) lymphoma of the lung is good. The 10-year survival rate is over 70%.^2^

Lymphoid organs are commonly classified as primary (or central), such as the thymus and bone marrow, or peripheral secondary lymphoid organs (e.g., lymph nodes, spleen, tonsils, Peyer’s patches); and are primarily responsible for inducing local immune responses. Tertiary lymphoid tissues (TLTs), on the other hand, are inducible ectopic lymphoid portions that develop de novo at sites of chronic inflammation in nonlymphoid organs.

**Figure 4.**
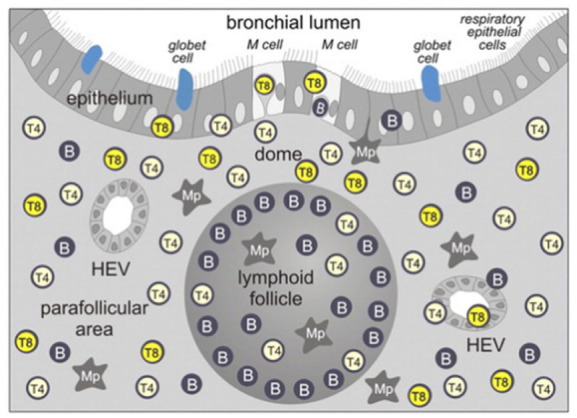
Schematic drawing of BALT in distribution of lymphocyte subsets is shown as in BALT of children. Three main compartments are: (1) Central follicular vein with accumulation of B lymphocytes. (2) The parafollicular area with mainly T lymphocytes and specialized HEV and (3) the dome area with a specialized epithelium lacking goblet cells. Various efferent and afferent autonomic nerves are indicated. There are efferent lymph vessels that drain to the regional parabronchial lymph nodes (with permission by Pabst^19^). HEV: high-endothelial venules.

Like lymph nodes, TLTs initiate adaptive immune responses and coordinate local tissue immunity. They form highly dynamic structural morphological entities ranging from lymphocyte aggregates to highly organized clusters with networks of follicular dendritic cells and germinal centers.^3^ They certainly share structural and functional features with conventional secondary lymphoid organs: T cell zones, B cell zones, marginal zones of antigen-presenting cells, reticular stromal networks, and high-endothelial veins (HEVs; Figure 4). However, the developed lymph node is quite different from the tertiary inducible lymphoid tissues in terms of plasticity. During ontogenetic lymph node development, maturation and accumulation of lymphoid progenitors from the mesoderm occur at more or less anatomical predilection sites, where germinal center structures form via HEV around preformed sinusoidal structures.^3^ In contrast, TLTs can induce a functional lymphoid aggregate within a very short time of a few hours. An important cell population that plays a regulatory role in the neogenesis of TLS is regulatory T cells (Tregs). An effective humoral and cellular immune response is based on primary (thymus, bone marrow) and secondary lymphoid organs (e.g. spleen, tonsils, lymph nodes).^3,4^ In 1973, John Bienenstock introduced the term BALT as part of MALT. All of his studies were performed in rabbits.^4,5^ In a morphologic study of lungs from many different species, the frequency of BALT varied widely, from 100% in rabbits to 30% in pigs and none in cats.^6^ In a study of many adult human lungs with a pathologic diagnosis of chronic bronchitis and bronchiectasis, BALT was found in 8% when the bronchus was obstructed by a tumor,^7^ whereas no BALT was found in 34 adult human lungs without inflammation.^6^ In the lungs of children who died of sudden infant death syndrome, 34% had BALT, and in those who died of other causes, 44% had BALT on histologic sections.^8,9^ In a recently published study,^10^ BALT structures were compared with pulmonary draining lymph nodes in children from birth to 13 years of age. The number of BALT decreased after 3 years of age, coinciding with an accumulation of memory T cells. B cells generated a skewed antibody response to several respiratory pathogens. Gould and Isaacson^11^ studied 102 fetal lungs and 12 infant lungs whereby fetal lungs showed BALT in 77% of cases and all cases of fetal lungs with BALT-like structures were associated with choriomeningitis or postpartum pneumonia.^11^ Lymphoid aggregates can be induced in structures known as inducible bronchus-associated lymphoid tissue (iBALT), which are often located in peribronchiolar or perivascular spaces. It is well documented that BALT is an obvious sign of chronic stimulation, for example, in smokers 82%, in nonsmokers 14%.^12^ In a more detailed study, Hogg et al. could show a correlation of BALT formation with the stage of chronic obstructive pulmonary disease (COPD).^13,14^ The severe stages of COPD have been shown to be associated with the emergence of remodeled and dendritic cell-rich alveolar–lymphoid interfaces. Although chronic antigen stimulation is generally associated with the formation of iBALT and MALT lymphoma^15^ by increasing the risk of lymphomatous transformation, there is controversial evidence for the role of bacterial and viral infections.^16^

In this study, we hypothesize a clinical evidence between the preceded severe SARS-CoV2-infection and the formation of the nodular lymphoid hyperplasia with the initial formation of a low-grade MALT lymphoma. The recently described SARS-CoV2-related systemic vascular inflammation and endothelial dysfunction^17,18^ is likely to contribute to this prolonged pulmonary inflammation by a recruitment of perivascular and peribronchial inducible BALT tissue^19^ accompanied by vascular alterations and the formation of intussusceptive angiogenesis. It has been demonstrated that angiogenesis and lymph angiogenesis play a pivotal role in the formation of blood tissue. The dynamics of flow and shear forces within HEV in newly formed blood vessels have been shown to be instrumental in the recruitment of new lymphocytes and their subsequent self-organization.^20,21^ It is evident that several questions have yet to be answered, including the nature of the cells that initiate the BALT anlagen, the contribution of epithelial-derived mediators and local DC networks, the role of T follicular helper cells in iBALT formation and function, and the mechanisms by which Tregs control iBALT organogenesis.^22^ The efficient priming of T cell responses directed against unrelated airborne antigens is a crucial process that requires dendritic cells for its sustained presence.^23^

## Conclusions

Further clinical investigations are needed to elucidate the role of viral and bacterial infections in the formation of BALT tissue and the involvement in the lymphomatous transformation of iBALT.

## References

[1] Sammassimo S Pruneri G Andreola G , et al A retrospective international study on primary extranodal marginal zone lymphoma of the lung (BALT lymphoma) on behalf of International Extranodal Lymphoma Study Group (IELSG). Hematol Oncol 2016; 34(4): 177–183. 26152851 10.1002/hon.2243

[2] Stefanovic A Morgensztern D Fong T , et al Pulmonary marginal zone lymphoma: a single centre experience and review of the SEER database. Leuk Lymphoma 2008; 49(7): 1311–1120. 10.1080/10428190802064933 18604720

[3] Brandtzaeg P Pabst R. Let’s go mucosal: communication on slippery ground. Trends Immunol 2004; 25(11): 570–577. Erratum in: *Trends Immunol* 2005; 26(1): 12. 15489184 10.1016/j.it.2004.09.005

[4] Brandtzaeg P Kiyono H Pabst R , et al Terminology: nomenclature of mucosa-associated lymphoid tissue. Mucosal Immunol 2008; 1: 31–37. 19079158 10.1038/mi.2007.9

[5] Bienenstock J McDermott MR. Bronchus- and nasal-associated lymphoid tissues. Immunol Rev 2005; 206: 22–31. 16048540 10.1111/j.0105-2896.2005.00299.x

[6] Pabst R Gehrke I. Is the bronchus-associated lymphoid tissue (BALT) an integral structure of the lung in normal mammals, including humans? Am J Respir Cell Mol Biol 1990; 3(2): 131–135. 2378747 10.1165/ajrcmb/3.2.131

[7] Delventhal S Brandis A Ostertag H , et al Low incidence of bronchus-associated lymphoid tissue (BALT) in chronically inflamed human lungs. Virchows Arch B Cell Pathol Incl Mol Pathol 1992; 62: 271–274. 1359700 10.1007/BF02899692

[8] Tschernig T Kleemann WJ Pabst R. Bronchus-associated lymphoid tissue (BALT) in the lungs of children who had died from sudden infant death syndrome and other causes. Thorax 1995; 50(6): 658–660. 7638809 10.1136/thx.50.6.658 PMC1021267

[9] Pabst R. Is BALT a major component of the human lung immune system? Immunol Today 1992; 13(4): 119–122. 1580992 10.1016/0167-5699(92)90106-H

[10] Matsumoto R Gray J Rybkina K , et al Induction of bronchus-associated lymphoid tissue is an early life adaptation for promoting human B cell immunity. Nat Immunol 2023; 24(8): 1370–1381. 37460638 10.1038/s41590-023-01557-3 PMC10529876

[11] Gould SJ Isaacson PG. Bronchus-associated lymphoid tissue (BALT) in human fetal and infant lung. J Pathol 1993; 169(2): 229–234. 8445488 10.1002/path.1711690209

[12] Richmond I Pritchard GE Ashcroft T , et al Bronchus associated lymphoid tissue (BALT) in human lung: its distribution in smokers and non-smokers. Thorax 1993; 48(11): 1130–1134. 8296257 10.1136/thx.48.11.1130 PMC464898

[13] Hogg JC Chu F Utokaparch S , et al The nature of small-airway obstruction in chronic obstructive pulmonary disease. N Engl J Med 2004; 350: 2645–2653. 15215480 10.1056/NEJMoa032158

[14] Mori M Andersson CK Svedberg KA , et al Appearance of remodelled and dendritic cell-rich alveolar-lymphoid interfaces provides a structural basis for increased alveolar antigen uptake in chronic obstructive pulmonary disease. Thorax 2013; 68(6): 521–531. 23412435 10.1136/thoraxjnl-2012-202879

[15] Suarez F Lortholary O Hermine O , et al Infection-associated lymphomas derived from marginal zone B cells: a model of antigen-driven lymphoproliferation. Blood 2006; 107(8): 3034–3044. 16397126 10.1182/blood-2005-09-3679

[16] Borie R Caro V Nunes H , et al No evidence for a pathogen associated with pulmonary MALT lymphoma: a metagenomics investigation. Infect Agent Cancer 2021; 16(1): 10. 33549143 10.1186/s13027-021-00351-w PMC7868019

[17] Mentzer SJ Ackermann M Jonigk D. Endothelialitis, microischemia, and intussusceptive angiogenesis in COVID-19. Cold Spring Harb Perspect Med 2022; 12(10): a041157. 10.1101/cshperspect.a041157 PMC9524390 35534210

[18] Ackermann M Verleden SE Kuehnel M , et al Pulmonary Vascular Endothelialitis, Thrombosis, and Angiogenesis in Covid-19. N Engl J Med 2020; 383(2): 120–128. 32437596 10.1056/NEJMoa2015432 PMC7412750

[19] Pabst R. The bronchus-associated-lymphoid tissue (BALT) an unique lymphoid organ in man and animals. Ann Anat 2022; 240: 151833. 34670121 10.1016/j.aanat.2021.151833

[20] Baluk P Adams A Phillips K , et al Preferential lymphatic growth in bronchus-associated lymphoid tissue in sustained lung inflammation. Am J Pathol 2014; 184(5): 1577–1592. 24631179 10.1016/j.ajpath.2014.01.021 PMC4005985

[21] Blanchard L Girard JP. High endothelial venules (HEVs) in immunity, inflammation and cancer. Angiogenesis 2021; 24(4): 719–753. 33956259 10.1007/s10456-021-09792-8 PMC8487881

[22] Foo SY Phipps S. Regulation of inducible BALT formation and contribution to immunity and pathology. Mucosal Immunol 2010; 3(6): 537–544. 20811344 10.1038/mi.2010.52

[23] Halle S Dujardin HC Bakocevic N , et al Induced bronchus-associated lymphoid tissue serves as a general priming site for T cells and is maintained by dendritic cells. J Exp Med 2009; 206(12): 2593–2601. 19917776 10.1084/jem.20091472 PMC2806625

